# Image‐based and biochemical multimodal phenotyping for explainable classification of chia (*Salvia hispanica* L.) genotypes

**DOI:** 10.1002/jsfa.71038

**Published:** 2026-08-31

**Authors:** Sevim Akcura, Irem Poyraz, Beyza Ciftci, Mahmut Kaplan

**Affiliations:** ^1^ Department of Field Crops, Faculty of Agriculture Çanakkale Onsekiz Mart University Çanakkale Turkey; ^2^ Department of Field Crops, Faculty of Agriculture Erciyes University Kayseri Turkey

**Keywords:** *Salvia hispanica* L., machine learning, explainable artificial intelligence, multimodal phenotyping, image processing, genotype classification

## Abstract

**BACKGROUND:**

This study developed an explainable machine learning framework integrating morphological, color, and biochemical characteristics for classifying chia (*Salvia hispanica* L.) genotypes. A dataset was assembled from 1200 seed images spanning four genotypes, from which 17 morphological and color features were extracted. These were complemented by six sample‐level biochemical traits – crude protein, fat, ash, fiber, carbohydrate, and total sugar – obtained from the corresponding experimental‐unit seed sample, resulting in a total of 23 variables in the integrated dataset. The dataset was evaluated comparatively with 10 machine learning algorithms under repeated 10‐fold cross‐validation, with all preprocessing confined to each training fold to avoid data leakage.

**RESULTS:**

The highest performance was obtained with XGBoost, reaching 86.99% accuracy, a Matthews correlation coefficient of 0.820, a receiver operating characteristic (ROC) area of 0.975, and a precision–recall curve (PRC) area of 0.933; Simple Logistic followed closely at 86.85% accuracy, with comparable ROC and PRC areas (0.974 and 0.933). Significant differences among the algorithms were confirmed by the Friedman test (*P* = 2.47 × 10^−120^), with *post hoc* comparisons placing XGBoost and Simple Logistic within the same top‐performing group. Protein, fiber, ash, and fat were the most influential biochemical traits, while hue and saturation among color parameters and shape index and geometric mean diameter among morphological features also contributed appreciably. The G1 genotype, which showed comparatively high protein (27.62%) and fiber (40.62%) contents, was the most consistently distinguished class, with XGBoost and Simple Logistic achieving F‐measures of 0.954 and 0.955, respectively, whereas greater phenotypic overlap between G2 and G3 resulted in more frequent mutual misclassifications.

**CONCLUSION:**

These findings indicate that multimodal phenotyping, coupled with explainable machine learning, offers a practical and biologically interpretable decision‐support approach for chia genotype classification. © 2026 The Author(s). *Journal of the Science of Food and Agriculture* published by John Wiley & Sons Ltd on behalf of Society of Chemical Industry.

## INTRODUCTION

Chia (*Salvia hispanica* L.) has attracted increasing global interest as a functional food because of the nutritional and technological properties of its seeds. Chia seeds are rich in omega‐3 fatty acids, particularly *α*‐linolenic acid, and contain approximately 18–24% protein and 23–34% dietary fiber, together with polyphenols and other antioxidant compounds.^1^, ^2^, ^3^ Their soluble fiber, proteins, bioactive peptides, and phenolic compounds contribute to their nutritional and functional value.^1^, ^2^, ^4^, ^5^ Beyond direct consumption, chia seeds are increasingly used in baked products, meat analogs, and vegan formulations as gluten‐free texturizing agents, fat replacers, and antioxidant ingredients, supporting their growing importance for the food and nutraceutical industries.^2^, ^6^, ^7^

Accurate characterization of variation among chia genotypes is important for breeding, seed identification, and quality assessment. Genotypic variation can be expressed through seed characteristics such as coat color, shape, surface structure, and size, while differences in biochemical composition provide an additional dimension for genotype characterization.^8^ These complementary sources of phenotypic information may be particularly useful for distinguishing genotypes that cannot be reliably separated by visual characteristics alone. However, conventional seed classification based largely on visual inspection is labor‐intensive, operator‐dependent, and difficult to scale when large numbers of samples must be evaluated.^9^

Image processing provides a rapid and nondestructive approach for quantifying seed characteristics through image acquisition, segmentation, and extraction of morphological and color features.^10^ When combined with machine learning (ML), these quantitative descriptors can be used for automated seed classification. Algorithms including k‐nearest neighbors (kNN), decision trees, random forest, support vector machines (SVM), and gradient boosting methods have been applied to seed classification in different crops.^11^, ^12^ Conventional ML approaches are particularly relevant to medium‐sized datasets because of their relatively modest computational requirements and their potential for greater interpretability compared with more data‐intensive architectures.^9^, ^11^, ^13^

ML has been applied to genotype‐ and variety‐level seed classification in crops including ornamental pumpkin,^14^ snack‐type watermelon,^15^ maize,^16^ and forage grass.^17^ However, comparable studies focusing specifically on chia seed genotypes remain limited. Moreover, classification studies often emphasize overall predictive performance, providing less information on genotype‐specific misclassification patterns and the variables contributing to model decisions.^15^, ^16^ Feature importance and permutation importance analyses can complement conventional performance metrics by identifying the morphological, color, and biochemical variables associated with genotype discrimination. Comparative evaluations of multiple ML algorithms within the same chia seed dataset also remain scarce.^18^, ^19^, ^20^

Against this background, the study presented here evaluated an integrated phenotyping approach for the classification of chia seed genotypes. Morphological and color traits measured from individual seed images were combined with biochemical traits obtained from the corresponding experimental‐unit seed sample, and 10 ML algorithms were compared using repeated stratified 10‐fold cross‐validation. Model performance was evaluated at both overall and genotype‐specific levels using multiple classification metrics, confusion matrices, receiver operating characteristic (ROC) curves, and precision–recall curves (PRCs). Feature importance and permutation importance analyses were further used to examine which phenotypic variables contributed most strongly to genotype discrimination. It was hypothesized that integrating complementary morphological, color, and biochemical information would provide useful discriminatory information for chia genotype classification.

## MATERIALS AND METHODS

### Plant material and experimental design

In the present study, four *Salvia hispanica* L. (chia) genotypes (G1, G2, G3, and G4) were evaluated as plant material. Specifically, the seeds were produced during the 2023 and 2024 growing seasons in field trials conducted at the Medicinal and Aromatic Plants Center of the Balıkesir Metropolitan Municipality (approximately 39.503° N, 26.980° E; ~15 m.a.s.l.), Turkey. Seedlings of the chia genotypes were raised in 200 cm^3^ pots filled with a growing medium composed of 20% perlite, 30% peat, and 50% soil (by volume) under fully controlled greenhouse conditions until they reached a height of approximately 20 cm. In both growing seasons, seedlings were transplanted to the field during the first week of April according to a randomized complete block design with three replications (blocks). Within each block, the four genotypes were randomly assigned to plots, resulting in a total of 12 plots per season. Each plot (2 m × 12.5 m) consisted of four rows with 25 plants per row, spaced 50 cm apart both between and within rows, thus comprising 100 plants per plot. Seven days after transplanting, a basal fertilization of 60:60:60 kg ha^−1^ N:P:K was applied using a compound fertilizer (15:15:15). During the flowering stage, an additional 30 kg ha^−1^ N was supplied as urea. To meet the water requirements of the plants, drip irrigation was applied seven times in total during each growing season, with approximately 40 mm of water per square meter at each application. The soil of the experimental site was slightly alkaline (pH 7.4), with low salinity (<4 dS m^−1^), a sandy loam texture, and an organic matter content of 0.7%. Soil analyses indicated that the site was deficient in available phosphorus, potassium, and total nitrogen, whereas the micronutrient levels were sufficient to support plant growth. At harvest, to eliminate border effects, the two outer rows of each plot were excluded, and only the 50 plants in the two central rows were considered. From these, 40 plants per plot were selected and hand‐harvested for seed collection. The harvested seeds were cleaned manually, and the seeds obtained in both experimental years were stored at −18 °C. These seeds constituted the plant material of the present study. For the image‐processing analyses, an equal number of seeds were sampled from each experimental unit of each genotype across the two growing seasons. For each genotype, 50 seeds were selected from each of the three field replications in each season, resulting in 300 individual seeds per genotype (2 seasons × 3 replications × 50 seeds) and 1200 seeds across the four genotypes. For the biochemical analyses, 100 g of seed was retained from each corresponding genotype × season × replication experimental unit, resulting in a total of 600 g of seed material per genotype (2 seasons × 3 replications × 100 g). Importantly, the 50 seeds used for image analysis from each experimental unit were selected from the same 100 g seed sample used for the corresponding biochemical analyses. This sampling structure preserved the direct link between each individual seed image and the biochemical profile of its source experimental unit.

To keep the analysis reliable, only seeds that were structurally intact, free of physical deformation, and morphologically homogeneous were retained. Because the same number of seeds was drawn from each genotype, the four classes were represented equally, so the dataset was balanced by design rather than through any resampling procedure. The overall workflow of the proposed image‐based and biochemical multimodal phenotyping framework is summarized in Fig. 1. Model performance was subsequently assessed through the repeated stratified cross‐validation scheme described in Section Cross‐validation strategy and data partitioning, with the class proportions preserved in every fold.

**Figure 1:**
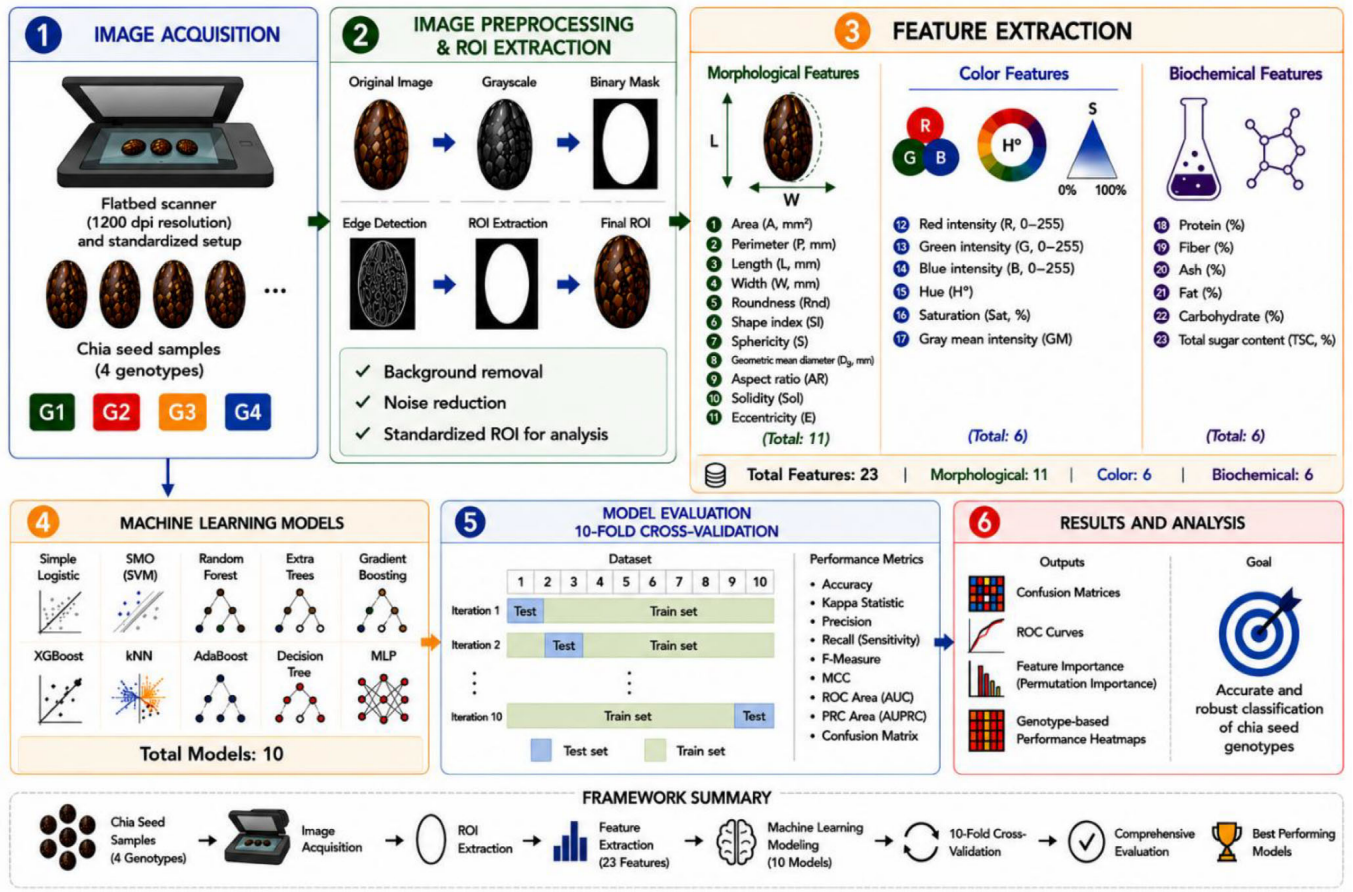
Overall workflow of the ML framework used for chia seed genotype classification. The workflow includes image acquisition and preprocessing, individual seed ROI extraction, morphological and color feature extraction, integration of biochemical variables, ML model development, repeated 10‐fold cross‐validation, statistical comparison, and model performance evaluation.

### Image acquisition and dataset preparation

A high‐resolution flatbed scanner system was used to acquire digital images of the seed samples. To minimize technical variation during image acquisition, all seeds were scanned using the same flatbed scanner settings and background conditions. To capture morphological and textural features with maximum accuracy, the images were scanned at 1200 dpi (dots per inch) optical resolution and transferred to the digital environment in PNG (Portable Network Graphics) format, avoiding lossy compression algorithms. Throughout the imaging protocol, a strategy to minimize technical variations was adopted; accordingly, software correction functions such as automatic brightness, contrast, and color calibration were disabled. This standardized acquisition and processing protocol was used to minimize technical variation associated with image acquisition and processing, thereby improving the consistency of morphological and color measurements across genotypes. The dataset was organized using a genotype‐specific hierarchical folder architecture, and each seed image was digitally matched with its corresponding genotype tag.

### Region of interest (ROI) extraction and image processing pipeline

For each genotype, 300 seeds originating from the six experimental units (2 seasons × 3 replications × 50 seeds) were arranged on the scanner surface and captured simultaneously in a single high‐resolution scan at 1200 dpi. Thus, the 1200 seeds representing the four genotypes were acquired in four genotype‐specific scans rather than being scanned individually. Experimental‐unit identities were retained during ROI extraction and data integration. A representative raw scan containing 300 seeds is shown in Fig. 2(A). Each high‐resolution image was subsequently processed to detect and separate the individual seeds, resulting in 300 individual seed images (ROIs) from each genotype‐specific scan.

**Figure 2:**
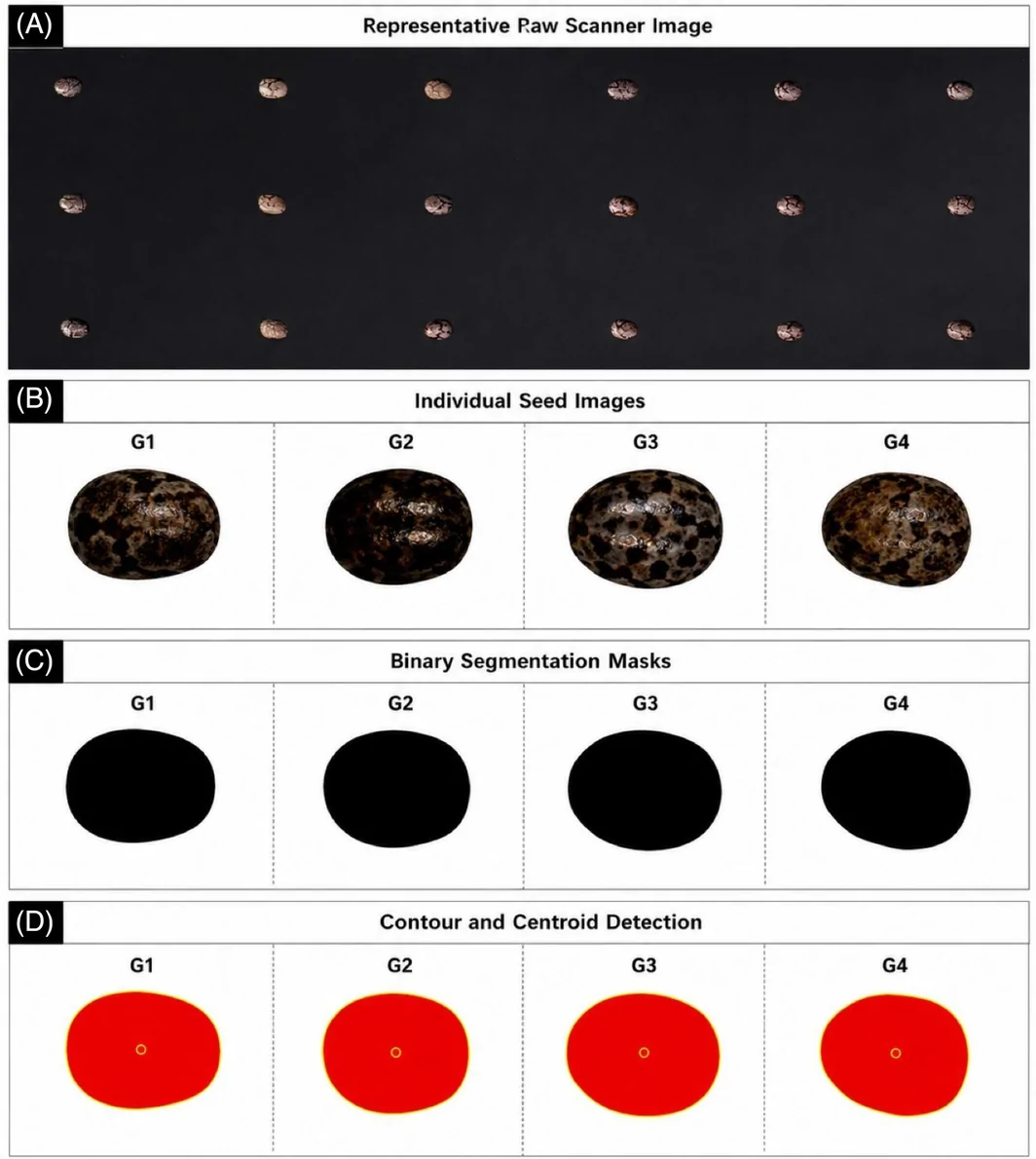
Image acquisition, preprocessing, and segmentation workflow used for chia seed genotype analysis. (A) Representative 1200 dpi raw scan showing the simultaneous arrangement of 300 chia seeds from a single genotype. (B) Representative individual seed images of the four chia genotypes (G1–G4). (C) Binary segmentation masks obtained after grayscale conversion and threshold‐based segmentation. (D) Detected seed contours and centroids used for subsequent extraction of morphological and color features.

Following image acquisition, the original RGB images were converted to grayscale and Gaussian filtering was applied to reduce high‐frequency noise before segmentation. Seeds were separated from the background using threshold‐based segmentation, followed by morphological operations to refine the resulting binary masks. Connected‐component and contour analyses were then used to identify individual seed regions. Each detected seed was individually extracted as a ROI based on its contour boundaries. The resulting individual‐seed ROIs were used for subsequent morphological and color feature extraction. The same preprocessing and ROI extraction procedure was applied uniformly to all four genotype‐specific scans.

### Morphological, color, and biochemical feature extraction

A comprehensive set of morphological, color, and biochemical variables was used to characterize the phenotypic profiles of the chia seeds. Eleven morphological descriptors were measured from individual seed images: area, perimeter, length, width, roundness, shape index, sphericity, geometric mean diameter, aspect ratio, solidity, and eccentricity. Color was characterized using the mean red, green, and blue intensities of the RGB color space, hue and saturation from the HSV color space, and the mean gray‐level intensity of the seed surface. Six biochemical traits were also included: crude protein, crude fiber, crude ash, crude fat, total carbohydrate, and total sugar content. Protein was quantified by the Kjeldahl method, fat by Soxhlet extraction, and ash and fiber gravimetrically.^21^, ^22^, ^23^, ^24^ Total carbohydrate was calculated by difference,^25^, ^26^ while total sugar content was determined by aqueous extraction followed by drying.^6^ All biochemical determinations followed the standard laboratory procedures cited above.

Morphological and color traits were measured directly at the individual‐seed level, whereas biochemical traits were determined for the corresponding 100 g seed sample from each genotype × season × replication experimental unit. For each genotype, six such experimental‐unit samples were analyzed (2 seasons × 3 replications), corresponding to a total of 600 g of seed material. The biochemical values obtained for each experimental unit were linked to the 50 individual‐seed records selected from that same 100 g sample. No interpolation, partitioning, or adjustment of the biochemical values according to individual seed mass was performed. For seed *j* originating from replication *k* of genotype *i* in season *s*, the integrated feature vector was defined as *X*_*iskj*_ = [*M*_*iskj*_, *C*_*iskj*_, *B*_*isk*_], where *M*_*isk*j_ and *C*_*iskj*_ denote the morphological and color features measured directly from the individual seed, respectively, and *B*_*isk*_ denotes the biochemical profile of the corresponding experimental‐unit seed sample. Thus, morphological and color variables were measured at individual‐seed resolution, whereas biochemical variables represented the corresponding genotype × season × replication experimental unit. The morphological, color, and biochemical variables were subsequently integrated into a 23‐variable input matrix for ML analysis, allowing complementary image‐derived and compositional information from the corresponding experimental‐unit seed sample to be evaluated within the same classification framework.

### Machine learning algorithms

Ten ML algorithms were compared for the classification of the chia genotypes: Random Forest (RF), Extremely Randomized Trees (Extra Trees), Gradient Boosting (GB), AdaBoost, eXtreme Gradient Boosting (XGBoost), Decision Tree (DT), kNN, Multilayer Perceptron (MLP), a SVM trained with sequential minimal optimization (SMO‐SVM), and Simple Logistic regression. These models were chosen for their balance of classification accuracy, computational efficiency, and interpretability; classical (shallow) learners were preferred in particular because they are well suited to medium‐sized tabular datasets and offer comparatively straightforward interpretation of feature contributions.

Rather than relying on an automated search, each algorithm was configured with a fixed set of hyperparameters guided by preliminary trials and commonly recommended values – for example, 500 estimators for the tree‐based ensembles, 7 distance‐weighted neighbors for kNN, and a maximum depth of 4 with a learning rate of 0.03 for XGBoost. Where supported by the implementation, class‐weighting settings were retained; because the dataset was balanced by design, these did not alter class representation. All modeling was performed in Python using NumPy 2.0.2, pandas 2.2.2, OpenCV 4.13.0, Matplotlib 3.10.0, SciPy 1.16.3, scikit‐learn 1.6.1, and XGBoost 3.2.0.

### Cross‐validation strategy and data partitioning

Model performance was assessed with a repeated stratified 10‐fold cross‐validation scheme, chosen to give an objective and stable estimate of how well each algorithm would generalize. Within a single repeat, the dataset was split into 10 equal folds with the class proportions preserved; nine of these were used to train the model and the tenth was held out for testing. This cycle was then repeated 10 times, each time with a different random partition, so that every algorithm was ultimately evaluated over 100 train–test combinations rather than a single split.

Keeping the folds stratified ensured that the class balance of the full dataset was mirrored in every training and test set. Guarding against data leakage was equally important: all data‐dependent steps, such as scaling and imputation, were fitted on the training fold alone and only then applied to the corresponding test fold, never the other way around. Finally, a fixed random seed was used throughout, so that the entire procedure could be reproduced exactly.

### Feature importance and explainability analysis

To move beyond predictive accuracy and interpret which seed characteristics drove the classifications, two complementary importance analyses were carried out.

For the models that expose an internal importance measure – the tree‐based ensembles (RF, Extra Trees, Gradient Boosting, AdaBoost, DT, and XGBoost) and the Simple Logistic model – feature importances were obtained directly from the fitted estimators. For the tree‐based methods these correspond to the impurity‐based (Gini) importance scores, whereas for the logistic model the magnitudes of the standardized coefficients were used as importance indicators.

For the remaining models, which do not provide an intrinsic importance measure (kNN, SMO‐SVM, and MLP), permutation importance was computed instead. In this approach, each feature was randomly shuffled 20 times and the resulting drop in classification accuracy was recorded, so that a larger decrease reflects a more influential feature. Together, these two analyses identified the morphological, color, and biochemical features that contributed most to distinguishing the chia genotypes and made the decision behavior of the models more transparent.

### Performance evaluation metrics

The predictive ability of the models was judged not by a single number but by a broad set of complementary metrics. Overall correctness was captured through accuracy, precision, recall (sensitivity), and the F1 score, while the Kappa statistic and the Matthews correlation coefficient (MCC) – both more resistant to class imbalance – were added to give a more conservative picture of agreement. To describe performance independently of any fixed decision threshold, the area under the ROC curve (ROC‐AUC) and the area under the PRC (PRC‐AUC) were also reported.

Beyond these, the quality of the predicted class probabilities was examined through the mean absolute error and the root mean squared error, together with their relative counterparts (relative absolute error and root relative squared error), which express the deviation of the predicted probabilities from the true class indicators against a class‐prior baseline. Finally, class‐level behavior was resolved for each genotype using the counts of true positives, true negatives, false positives, and false negatives, from which the per‐class precision, recall, F‐measure, MCC, ROC‐AUC, and PRC‐AUC were derived.

### Statistical analysis

The statistical evaluation addressed two separate questions: how the four genotypes differed in their measured traits, and how the 10 classification algorithms compared in predictive performance.

Genotypic variation was examined by subjecting each morphological, color, and biochemical trait to one‐way ANOVA. Where the genotype effect proved significant, mean values were separated using Fisher's least significant difference (LSD) test at *P* < 0.05. Trait values are reported as mean ± standard deviation, and within each row of Table 1, genotypes not sharing a common letter differ significantly. The biochemical measurements are expressed on a dry‐weight basis. These analyses were performed in JMP 18 (SAS Institute Inc., Cary, NC, USA).

**Table 1:** Morphological, color, and biochemical features used in ML analysis

Trait	G1	G2	G3	G4
Area (A, mm^2^)	1.623 ± 0.250 ^a^	1.364 ± 0.265 ^d^	1.454 ± 0.225 ^b^	1.410 ± 0.214 ^c^
Perimeter (P, mm)	5.575 ± 0.447 ^a^	5.260 ± 0.441 ^c^	5.460 ± 0.451 ^b^	5.246 ± 0.408 ^c^
Length (L, mm)	1.894 ± 0.144 ^a^	1.718 ± 0.142 ^c^	1.820 ± 0.126 ^b^	1.800 ± 0.135 ^b^
Width (W, mm)	1.210 ± 0.102 ^a^	1.206 ± 0.172 ^a^	1.158 ± 0.097 ^b^	1.124 ± 0.101 ^c^
Roundness (Rnd)	0.573 ± 0.035 ^b^	0.588 ± 0.084 ^a^	0.556 ± 0.040 ^c^	0.552 ± 0.035 ^c^
Shape index (SI)	1.544 ± 0.200 ^b^	1.658 ± 0.324 ^a^	1.664 ± 0.302 ^a^	1.570 ± 0.168 ^b^
Sphericity (S)	12.871 ± 1.860 ^a^	11.885 ± 2.218 ^b^	11.819 ± 1.559 ^b^	11.313 ± 1.688 ^c^
Geometric mean diameter (Dg, mm)	1.433 ± 0.112 ^a^	1.312 ± 0.125 ^d^	1.356 ± 0.105 ^b^	1.336 ± 0.101 ^c^
Aspect ratio (AR)	1.571 ± 0.112 ^b^	1.453 ± 0.231 ^c^	1.579 ± 0.133 ^b^	1.608 ± 0.123 ^a^
Solidity (Sol)	0.928 ± 0.028 ^a^	0.909 ± 0.041 ^c^	0.910 ± 0.039 ^c^	0.921 ± 0.028 ^b^
Eccentricity (Ecc)	0.813 ± 0.024 ^b^	0.813 ± 0.056 ^b^	0.820 ± 0.028 ^a^	0.825 ± 0.022 ^a^
Red intensity (R, 0–255)	127.457 ± 8.098 ^a^	128.555 ± 16.866 ^a^	120.553 ± 7.945 ^c^	122.454 ± 7.622 ^b^
Green intensity (G, 0–255)	105.515 ± 7.263 ^b^	107.387 ± 16.000 ^a^	100.788 ± 7.498 ^c^	100.725 ± 6.720 ^c^
Blue intensity (B, 0–255)	98.460 ± 7.829 ^b^	101.249 ± 15.760 ^a^	96.650 ± 7.469 ^c^	94.448 ± 6.682 ^d^
Hue (H°)	29.863 ± 13.164 ^c^	36.631 ± 18.026 ^b^	43.507 ± 15.341 ^a^	31.581 ± 11.611 ^c^
Saturation (Sat, %)	68.299 ± 11.390 ^b^	70.825 ± 17.314 ^a^	65.752 ± 10.089 ^c^	69.034 ± 9.540 ^ab^
Gray mean intensity (GM)	111.274 ± 7.226 ^b^	113.019 ± 15.877 ^a^	106.230 ± 7.391 ^c^	106.511 ± 6.715 ^c^
Protein (%)	27.617 ± 1.295 ^a^	25.314 ± 1.318 ^c^	26.738 ± 1.295 ^b^	24.012 ± 1.376 ^d^
Fiber (%)	40.615 ± 1.853 ^a^	34.809 ± 2.266 ^c^	36.579 ± 2.517 ^b^	36.282 ± 2.615 ^b^
Ash (%)	7.667 ± 0.504 ^a^	6.187 ± 0.504 ^d^	6.906 ± 0.608 ^b^	6.647 ± 0.523 ^c^
Fat (%)	37.726 ± 2.975 ^a^	29.021 ± 3.987 ^d^	33.598 ± 3.787 ^b^	29.740 ± 3.516 ^c^
Carbohydrate (%)	38.482 ± 4.649 ^d^	49.651 ± 6.062 ^b^	44.208 ± 6.292 ^c^	52.854 ± 6.195 ^a^
Total sugar content (TSC, %)	73.230 ± 5.507 ^a^	61.387 ± 5.130 ^c^	66.151 ± 5.530 ^b^	61.717 ± 5.411 ^c^

Values are presented as mean ± standard deviation for the four chia genotypes (G1–G4). Within each row, means followed by the same superscript letter are not significantly different according to Fisher's LSD test (*P* < 0.05); superscript letters are assigned in descending order of the mean (a denotes the highest value). The dataset comprises 23 features in total: 11 morphological (area, perimeter, length, width, roundness, shape index, sphericity, geometric mean diameter, aspect ratio, solidity, eccentricity), 6 color/intensity (red, green, blue, hue, saturation, gray mean intensity), and 6 biochemical (protein, fiber, ash, fat, carbohydrate, total sugar content) traits.

The comparison of algorithms called for a different approach, since the 10 models were evaluated repeatedly on the same data partitions. Each model was assessed through 10 repeats of stratified 10‐fold cross‐validation, yielding 100 accuracy estimates per algorithm, and these paired results were compared with the Friedman test, whose rank‐based structure suits repeated measurements of this kind. Because the test indicated that the algorithms did not perform equally (*P* < 0.05), it was followed by the Nemenyi *post hoc* test to identify which pairs differed. A mean rank was then assigned to each algorithm, with lower ranks reflecting better performance, and the resulting differences were visualized with a critical difference (CD) diagram (Fig. 3), in which algorithms whose mean‐rank differences exceeded the critical difference were considered significantly different. A fixed random seed was maintained across all runs to ensure reproducibility, and the statistical tests were carried out in Python using the SciPy and scikit‐posthocs libraries.

**Figure 3:**
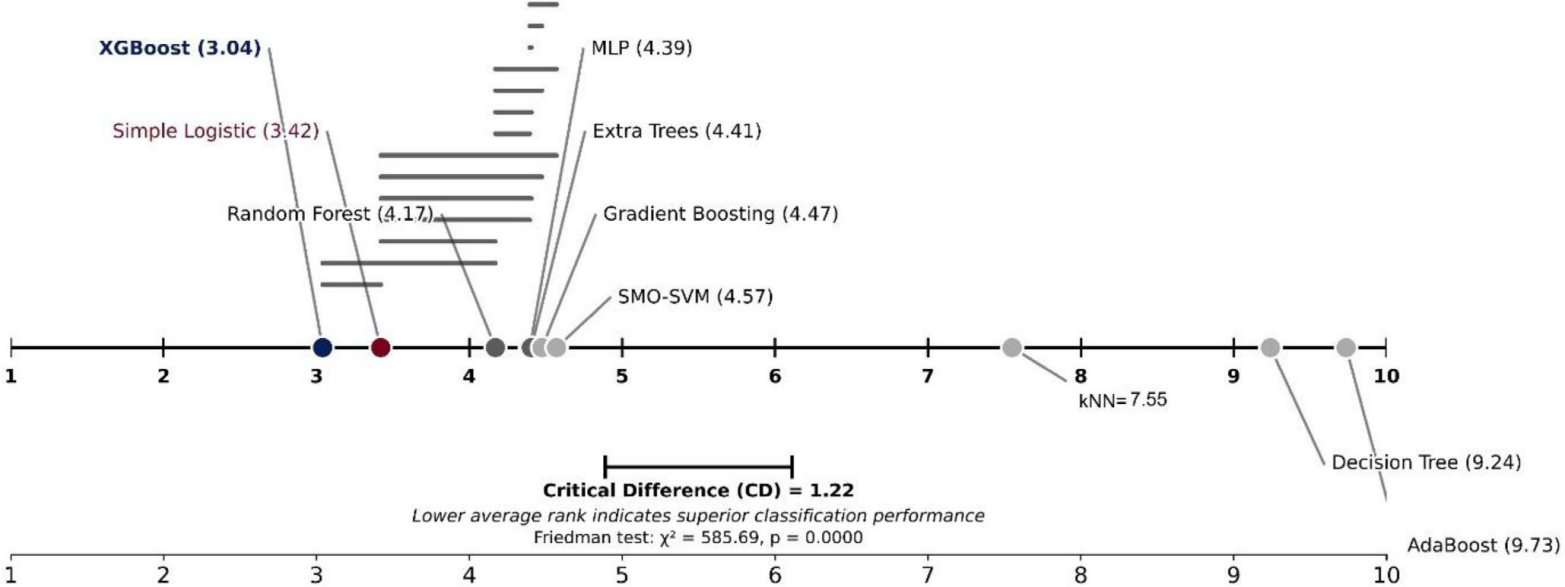
Friedman average‐rank and CD diagram comparing the performance of the ML algorithms across repeated classification experiments. Lower average ranks indicate better overall performance. XGBoost obtained the lowest numerical average rank, followed by Simple Logistic and RF; however, the *post hoc* Nemenyi test indicated no statistically significant differences among these upper‐ranked models. The critical difference (CD = 1.22) represents the minimum difference in average ranks required for statistical significance at the selected significance level. Horizontal bars connect algorithms whose performance did not differ significantly.

### Visualization

The results of the analytical process were illustrated through a set of high‐resolution figures. Confusion matrices were produced to display the error distribution of each classifier across the four genotypes, presented in row‐normalized (%) form so that per‐class accuracy could be read directly. ROC curves were then plotted for every model to compare their discriminative ability across genotypes. The traits driving the classifications were summarized as horizontal bar plots of feature importance for the models with an intrinsic importance measure and of permutation importance for the remaining models. Overall model performance was further condensed into a heatmap spanning eight evaluation metrics, while a second set of genotype‐specific heatmaps displayed the F‐measure, precision, recall, MCC, ROC‐AUC, and PRC‐AUC of each algorithm for every genotype, making class‐level strengths and weaknesses easy to compare at a glance. All figures were generated in Python with the Matplotlib library and exported at 600 dpi.

## RESULTS

### General classification performance of ML algorithms

When the overall classification performance of the 10 algorithms was examined, clear differences were observed among the models (Table 2; Table S1; Figs 4 and 5). XGBoost achieved the highest numerical accuracy (86.99%), closely followed by Simple Logistic (86.85%) and RF (85.95%). However, the *post hoc* Nemenyi test indicated no statistically significant differences among these three upper‐performing algorithms, showing that the numerical advantage of XGBoost should not be interpreted as statistical superiority over Simple Logistic or RF. A similarly close performance pattern was observed for the Kappa statistic and MCC. The MCC values were 0.820 for XGBoost, 0.822 for Simple Logistic, and 0.823 for RF (Table S1), further indicating comparable overall classification performance among these models.

**Table 2:** Statistical comparison of ML algorithms based on repeated cross‐validation, showing mean accuracy, Friedman mean ranks, and *post hoc* significance groups

Algorithm	Accuracy (mean ± SD)	Mean rank	Statistical group
XGBoost	0.8699 ± 0.0283	3.040	a
Simple Logistic	0.8685 ± 0.0277	3.420	ab
RF	0.8595 ± 0.0267	4.170	ab
Gradient Boosting	0.8578 ± 0.0306	4.475	b
Extra Trees	0.8566 ± 0.0288	4.405	b
MLP	0.8548 ± 0.0309	4.395	b
SMO‐SVM	0.8547 ± 0.0294	4.570	b
kNN	0.8132 ± 0.0311	7.550	c
DT	0.7230 ± 0.0402	9.240	d
AdaBoost	0.6946 ± 0.0350	9.735	d

Metric	Test	Statistic	*P*
Accuracy	Friedman test	585.688	2.47 × 10^−120^

Accuracy values are reported as mean ± standard deviation obtained from 10 repeats of 10‐fold cross‐validation (100 observations per algorithm). Mean ranks were derived from the Friedman test (*χ* ^2^(9) = 585.688, *P* = 2.47 × 10^−120^), which indicated statistically significant differences among the algorithms. Pairwise *post hoc* comparisons were performed using the Nemenyi test; algorithms sharing the same letter in the ‘statistical group’ column are not significantly different at *P* < 0.05. Lower mean ranks indicate superior classification performance.

**Figure 4:**
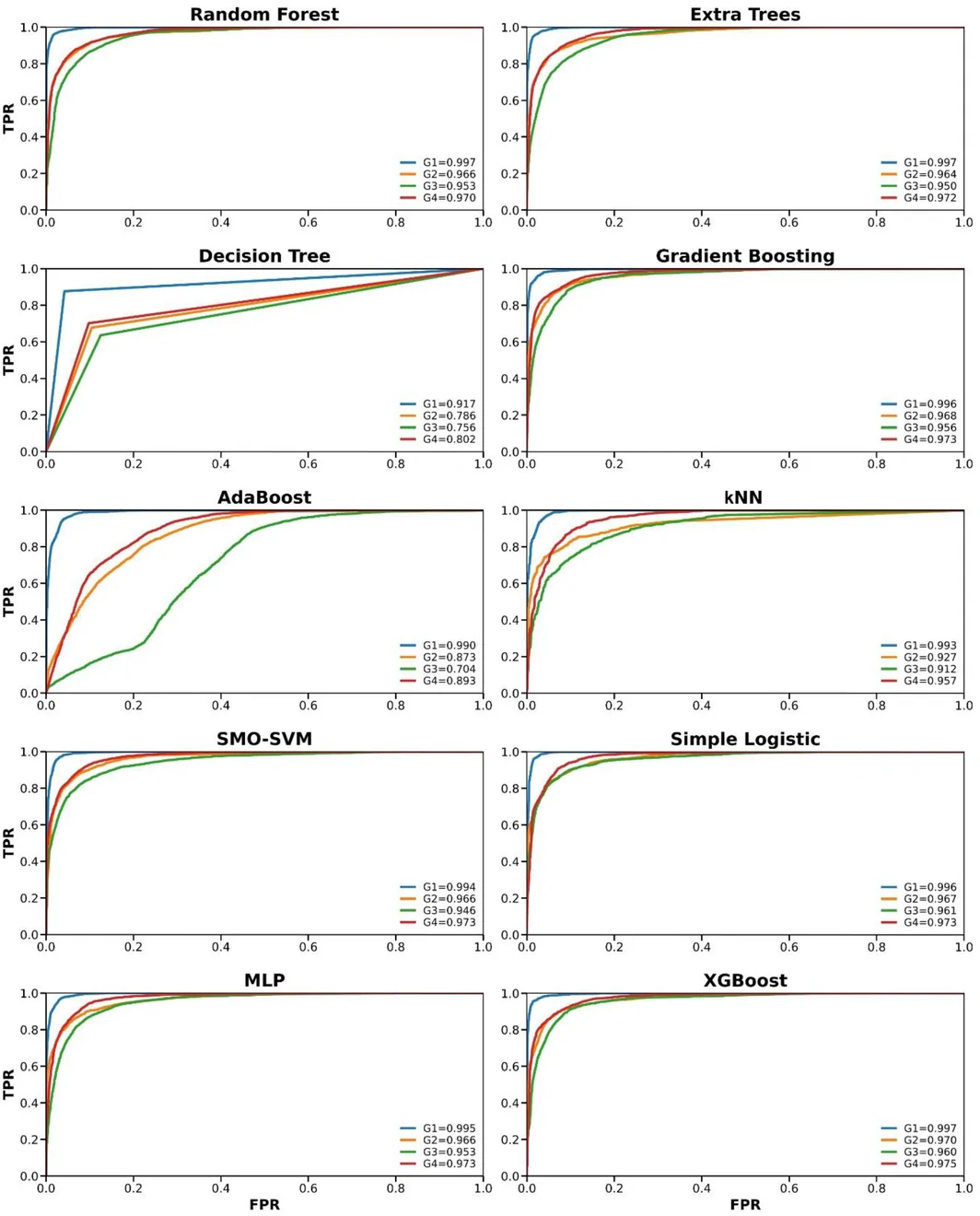
ROC curves and AUC values for ML‐based classification of chia seed genotypes. ROC curves and corresponding AUC values obtained for the 10 ML algorithms evaluated using repeated 10‐fold cross‐validation. The curves show the class‐specific discrimination performance of each algorithm across the four chia genotypes (G1–G4).

**Figure 5:**
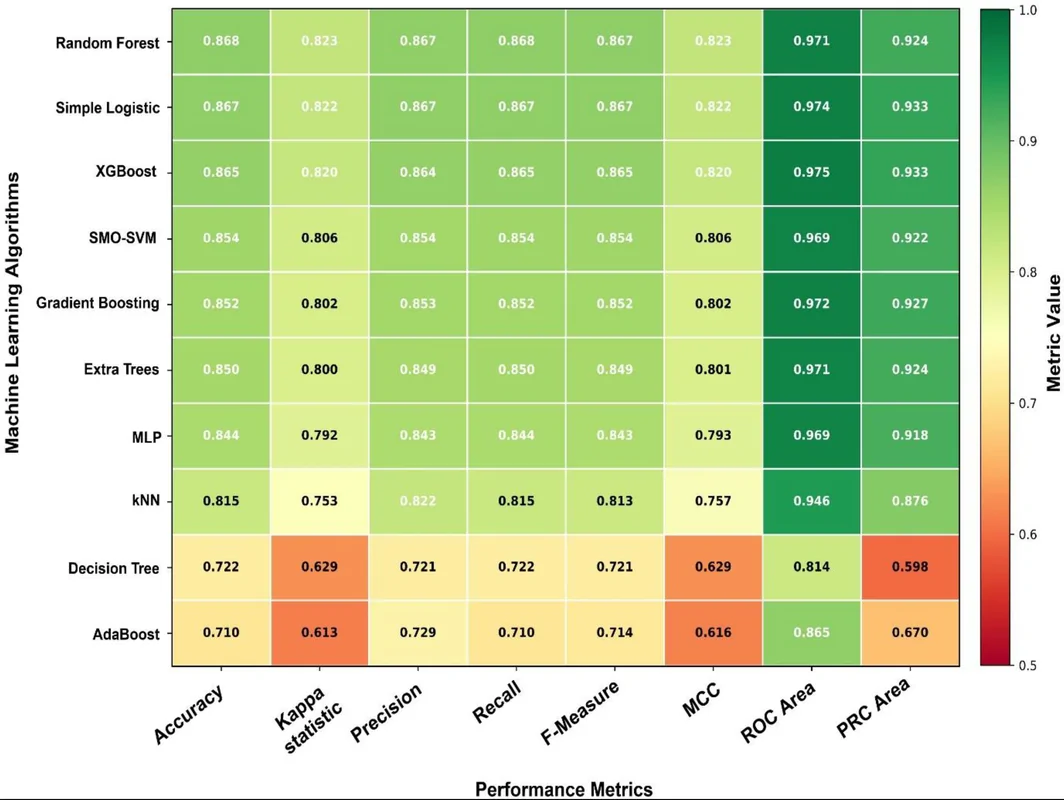
Heatmap of overall classification performance across ML algorithms and evaluation metrics. Heatmap showing the performance of the evaluated ML algorithms based on eight metrics: accuracy, Kappa statistic, precision, recall, F‐measure, MCC, ROC area, and PRC area. Darker green shading indicates higher metric values.

The leading models showed strong discriminative performance. The highest ROC area was attained by XGBoost (0.975), and closely similar values were produced by Simple Logistic (0.974), Gradient Boosting (0.972), Extra Trees (0.971), and RF (0.971) (Table S1; Fig. 4). These results were reinforced by the PRC area, for which XGBoost and Simple Logistic again stood out at 0.933. At the opposite end of the scale, the weakest overall performance was shown by DT and AdaBoost, whose accuracies were 72.30% and 69.46% and whose ROC areas were 0.814 and 0.865, respectively (Table 2; Table S1).

As can be seen from the performance heatmap in Fig. 5, consistently high scores were produced by XGBoost, Simple Logistic, and RF across accuracy, MCC, ROC area, and PRC area, whereas markedly larger fluctuations between metrics were observed for DT and AdaBoost; this pattern suggested that more stable classification was provided by the ensemble‐based algorithms. These differences were statistically confirmed by the Friedman test (*χ* ^2^ = 585.69; *P* = 2.47 × 10^−120^) (Table 2; Fig. 3). In the corresponding CD diagram (Fig. 3; CD = 1.22), the lowest average rank was obtained by XGBoost (3.04), which formed the top statistical group (a), while Simple Logistic (3.42) and RF (4.17) were placed in an overlapping group (ab); accordingly, these three leading models could not be clearly separated from one another. At the other extreme, the lowest‐ranked group was shared by DT (9.24) and AdaBoost (9.73). Taken together, these findings showed that XGBoost, Simple Logistic, and RF provided the strongest overall classification performance, with no statistically significant differences among these three upper‐performing models.

### Genotype‐based (class‐level) classification results

Class‐level analyses revealed that some genotypes were distinguished far more readily than others (Table S2; Figs 6 and 7). Across all models, the highest classification success was obtained for the G1 genotype. In the Simple Logistic model, for instance, precision, recall, and F‐measure values of 0.956, 0.955, and 0.955 were recorded for the G1 class, and comparable figures were produced by XGBoost, where a precision of 0.951, a recall of 0.958, and an F‐measure of 0.954 were obtained (Table S2). The ROC area for G1 exceeded 0.994 in all high‐performing models, indicating strong discrimination of G1 from the remaining genotypes.

**Figure 6:**
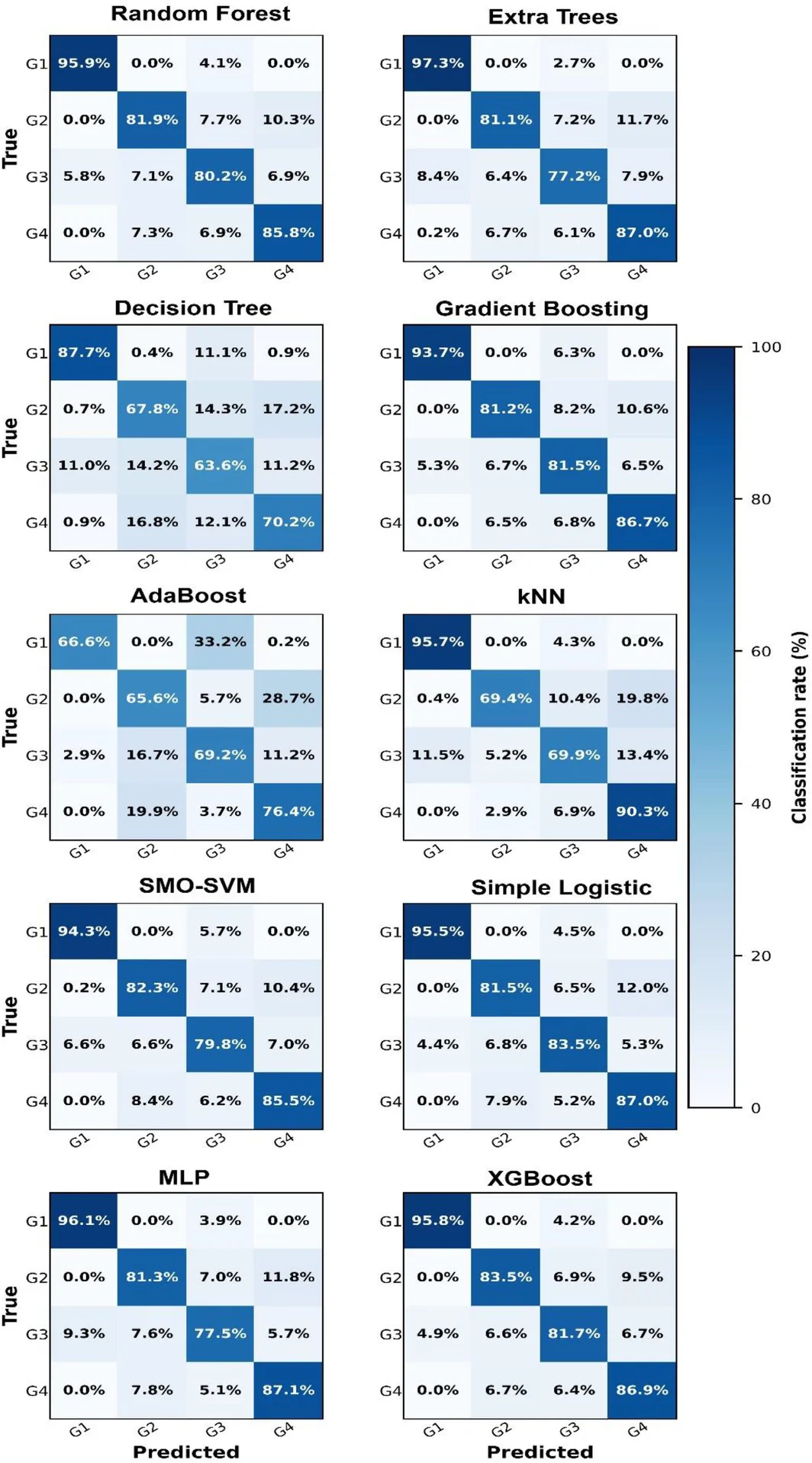
Normalized confusion matrices for ML‐based classification of chia seed genotypes. Normalized confusion matrices (%) obtained for the 10 ML algorithms evaluated using repeated 10‐fold cross‐validation. The matrices show genotype‐specific classification performance and misclassification patterns among the four chia genotypes (G1–G4).

**Figure 7:**
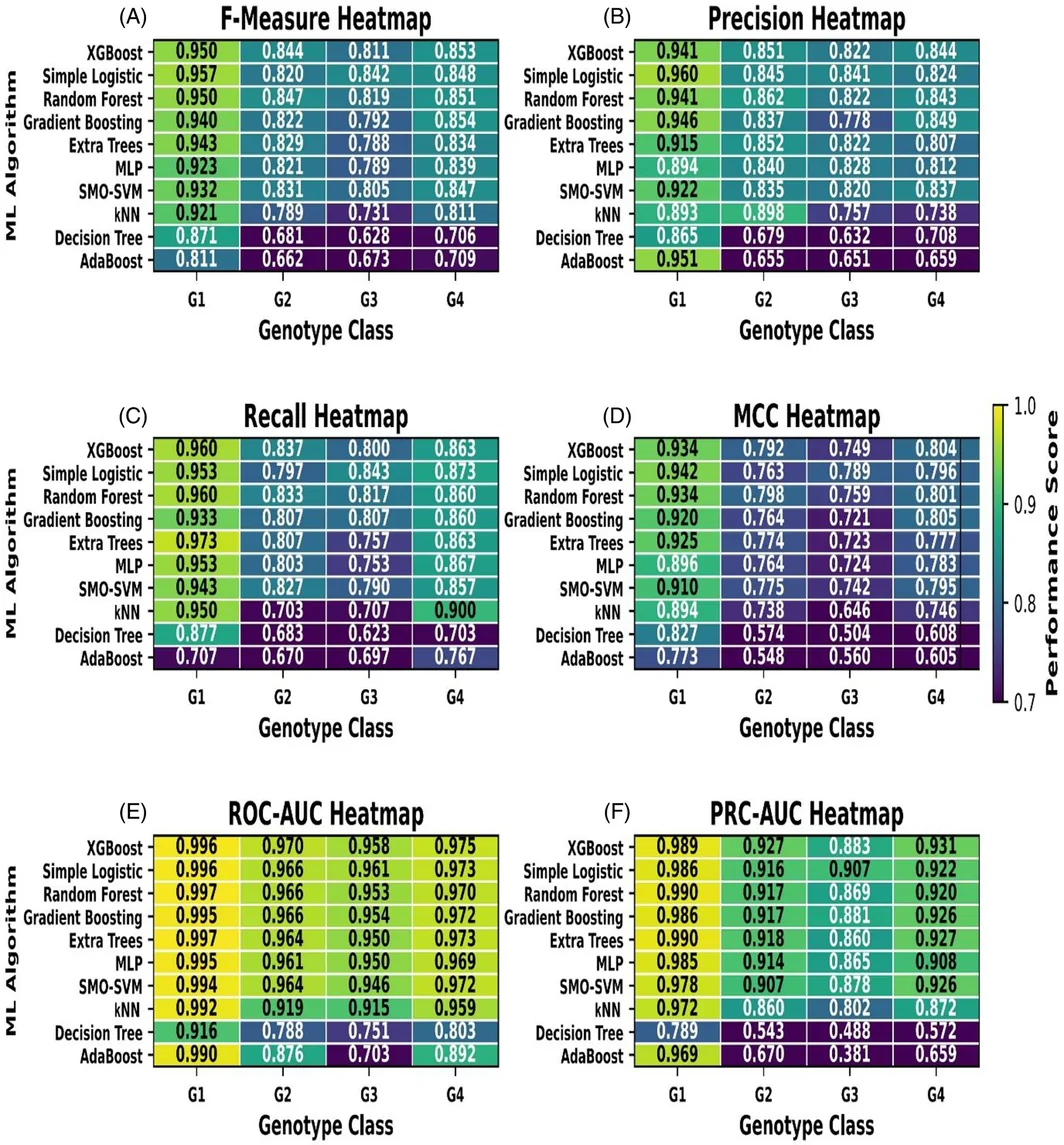
Heatmaps of genotype‐specific classification performance across ML algorithms. Genotype‐specific performance of the evaluated ML algorithms for the four chia genotypes (G1–G4), based on six evaluation metrics: (A) F‐measure, (B) precision, (C) recall, (D) MCC, (E) ROC‐AUC, and (F) PRC‐AUC. Higher color intensity indicates higher metric values.

A very different picture emerged for the G2 and G3 genotypes, between which a marked classification difficulty was observed. As the normalized confusion matrices in Fig. 6 show, the misclassifications were concentrated precisely between these two classes. In the RF model, recall values of 0.819 and 0.802 were obtained for G2 and G3, respectively, and similarly reduced F‐measures for these classes were seen in the Extra Trees and SMO‐SVM models relative to the other genotypes (Table S2). Taken together, these results suggest that G2 and G3 were more difficult to distinguish based on the integrated phenotypic information available to the models, including individual‐seed image traits and experimental‐unit‐level biochemical variables.

This confusion was found to become more pronounced in the weaker models. In the DT model, F‐measures of 0.681 and 0.633 were recorded for G2 and G3, while in the AdaBoost model these values fell to 0.649 and 0.654, respectively (Table S2). The drop of the PRC area for G3 to 0.376 in the AdaBoost model further indicated that this model possessed only limited capacity to distinguish that genotype. Intermediate discriminability was shown by the G4 genotype: among the strongest models – XGBoost, Gradient Boosting, and Simple Logistic – F‐measures of 0.856, 0.851, and 0.851 were obtained for the G4 class, and its ROC area ranged from 0.973 to 0.975, indicating that this class too could be predicted with high accuracy (Table S2).

The genotype‐based heatmaps in Fig. 7 make clear that the models' ability to separate the classes varied considerably from one genotype to another. When all metrics were considered together, the highest F‐measure, precision, recall, MCC, ROC‐AUC, and PRC‐AUC values were consistently associated with the G1 genotype across almost all algorithms, which indicated that G1 was the most consistently distinguished genotype across the evaluated models. Its comparatively distinct morphological, color, and experimental‐unit‐level biochemical profiles may have contributed to this pattern. Overall, more balanced and stable performance across genotypes was displayed by the XGBoost, Simple Logistic, RF, and SMO‐SVM algorithms, whereas weaker discrimination, particularly for the G2 and G3 classes, was characteristic of the DT and AdaBoost models.

### Feature importance and explainability findings

The feature importance analyses provided insight into the variables associated with model predictions and showed that several biochemical traits contributed to chia genotype classification (Fig. 8; Table 1). Protein, fiber, ash, and fat were among the higher‐ranked variables in several of the high‐performing models. As is evident from Table 1, G1 differed from the other genotypes in several biochemical variables, including protein, fiber, ash, and fat content. These compositional differences may have contributed to the consistently high classification performance observed for G1. Consistent with this, F‐measures of 0.954 and 0.955 were recorded for G1 in the XGBoost and Simple Logistic models, respectively (Table S2).

**Figure 8:**
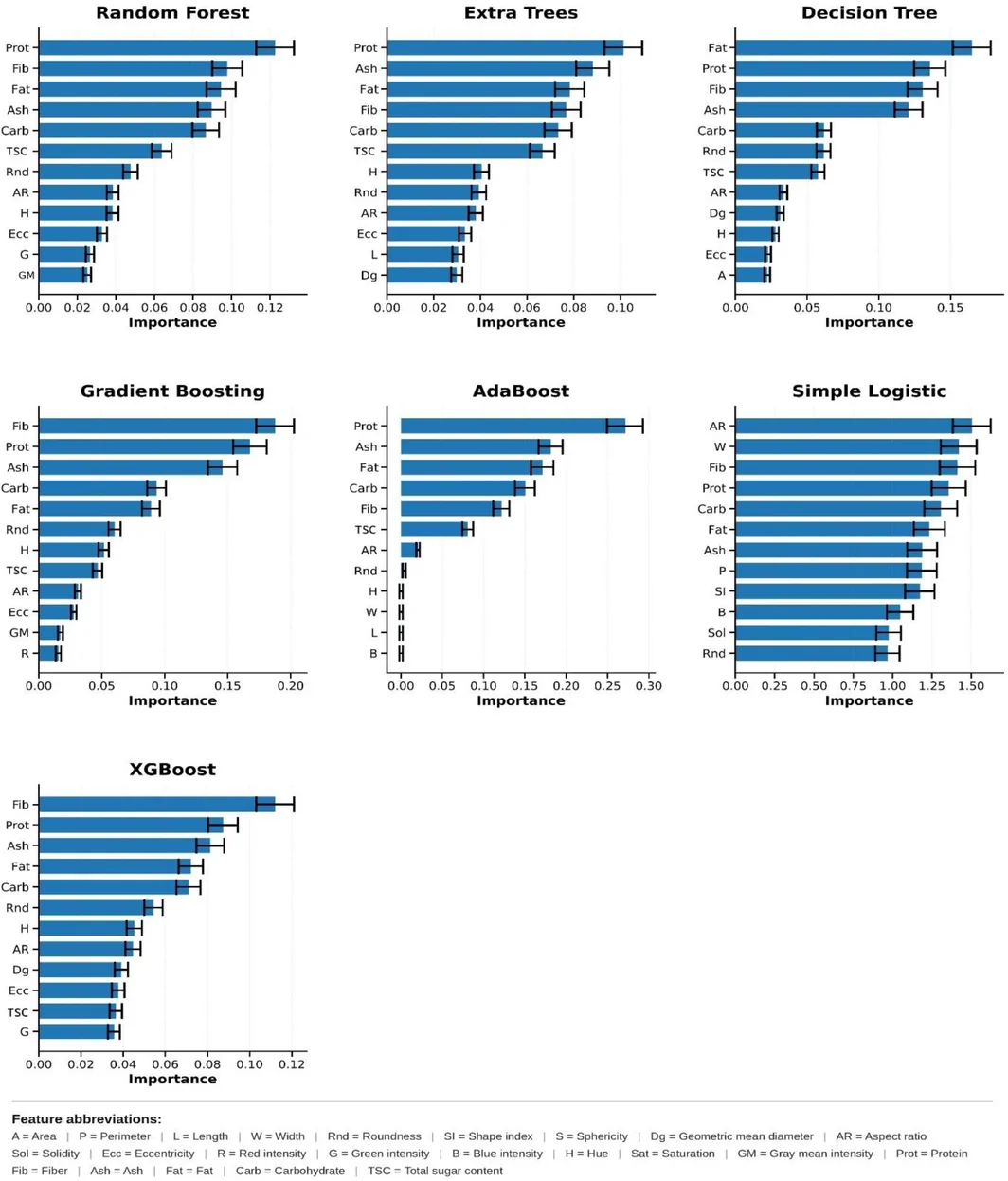
Model‐specific feature importance profiles for chia seed genotype classification. Feature importance profiles showing the relative contributions of morphological, color, and biochemical variables to chia seed genotype classification. For Simple Logistic, importance values represent the mean absolute coefficient magnitudes across classes obtained from standardized input features (approximately 0–1.50), whereas for the tree‐based models and XGBoost, the model‐specific feature importance scores returned by the fitted estimators are shown on substantially smaller numerical scales. Because these importance measures are model‐specific and are expressed on different numerical scales, their absolute values should not be directly compared across panels. Feature abbreviations are defined at the bottom of the figure.

The importance profiles in Fig. 8 clearly reflected that greater weight was assigned to protein, fiber, ash, and fat by the ensemble‐based algorithms. The overall accuracies of XGBoost, RF, and Gradient Boosting were 86.99%, 85.95%, and 85.78%, respectively (Table 2), while their ROC areas ranged from 0.971 to 0.975 (Table S1; Fig. 4), indicating strong overall classification and discrimination performance.

Morphological and color‐based features also contributed to genotype classification. As presented in Table 1, the highest hue value was recorded for G3 (43.507°), compared with 29.863° for G1, while the highest saturation was observed for G2 (70.825%). Consistent with these differences, color‐related variables appeared among the influential features in the model‐specific importance profiles (Fig. 8). Morphological descriptors, including shape index, roundness, and geometric mean diameter, also contributed to model predictions. For example, G1 had a geometric mean diameter of 1.433 mm and a sphericity value of 12.871 (Table 1). Together, these results indicate that image‐derived morphological and color characteristics provided complementary information for distinguishing the chia genotypes alongside the biochemical variables.

### Permutation importance analyses

The permutation importance analyses are presented in Fig. 9 and provide a complementary view of the variables contributing to predictions by kNN, SMO‐SVM, and MLP. Several biochemical variables, together with morphological and color‐related features, appeared among the higher‐ranked variables across these models.

**Figure 9:**
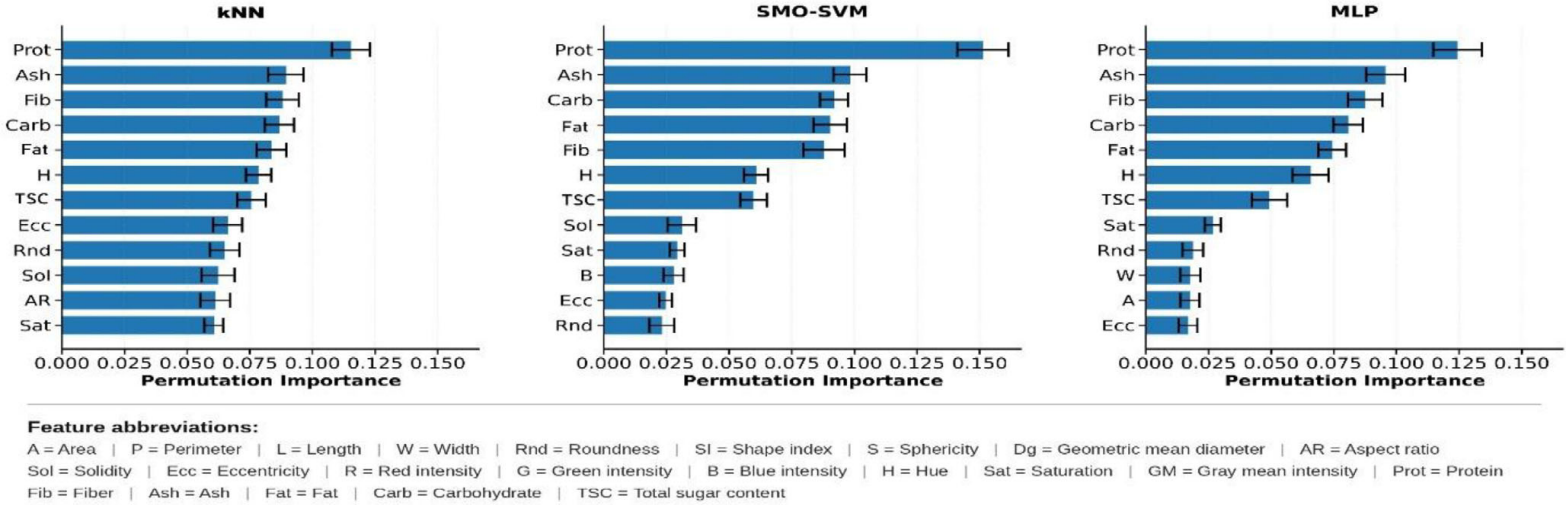
Permutation importance profiles for kNN, SMO‐SVM, and MLP models in chia seed genotype classification. Permutation importance profiles showing the contributions of morphological, color, and biochemical variables to genotype classification. Importance values represent the mean decrease in classification accuracy after permutation of each variable. The same permutation procedure and performance metric were used for kNN, SMO‐SVM, and MLP, allowing the resulting importance values to be compared across the three models. Feature abbreviations are defined at the bottom of the figure.

For the kNN model, pronounced decreases in accuracy were observed when protein, fiber, and ash were permuted, indicating that these variables contributed substantially to the model's predictions. This dependence was consistent with its overall accuracy of 81.32% and MCC of 0.757 (Table S1 and Table 2); moreover, the fall in recall to 0.694 and 0.699 for the G2 and G3 classes, respectively, indicated that phenotypically similar genotypes were difficult for the model to separate (Table S2; Fig. 6).

For the SMO‐SVM model, protein, ash, and carbohydrate were among the most influential features (Fig. 9). An overall accuracy of 85.40%, a MCC of 0.806, and a ROC area of 0.969 were obtained for this model (Table S1). For G1, the model achieved an F‐measure of 0.938 and a ROC area of 0.994 (Table S2), consistent with its strong discrimination of this genotype.

For the MLP model, high permutation importance was associated with protein, fiber, and ash content (Fig. 9). This model performed well overall, with an accuracy of 84.40%, a MCC of 0.793, and a ROC area of 0.969 (Table S1); nonetheless, the decrease of the G3 F‐measure to 0.801 indicated that phenotypically similar genotypes were only partially resolved by the model (Table S2).

The permutation importance profiles in Fig. 9 showed that several biochemical, morphological, and color‐related variables contributed to classification across models with different decision mechanisms. The recurrence of some of these variables among the higher‐ranked features in Fig. 8 suggests that complementary phenotypic dimensions contributed useful information to genotype discrimination. In particular, the relatively high importance assigned to several biochemical variables indicates that compositional differences among the corresponding experimental‐unit seed sample contributed to the discrimination of chia genotypes in the present dataset.

## DISCUSSION

This study showed that morphological, color‐based, and biochemical traits can be integrated for the classification of chia genotypes. High accuracy, MCC, ROC area, and PRC area values were obtained, particularly with XGBoost, Simple Logistic, and RF, indicating that complementary phenotypic information can support reliable genotype discrimination.^27^, ^28^

The combined evaluation of complementary phenotypic dimensions may provide a broader representation of variation among genotypes than any single feature group considered in isolation.^29^ Data‐driven phenotyping systems are also playing an increasingly important role in plant breeding programs.^30^

A notable outcome of this study was the consistently high performance of several algorithms with different learning structures. XGBoost achieved the highest numerical accuracy and the lowest mean rank in the Friedman analysis, while Simple Logistic and RF showed closely comparable performance. Importantly, however, the *post hoc* Nemenyi test indicated no statistically significant differences among XGBoost, Simple Logistic, and RF within the upper‐performing group. Thus, the numerical advantage observed for XGBoost should not be interpreted as statistical superiority over the other two models. The strong performance of RF and other tree‐based ensemble methods is consistent with previous reports showing that ensemble approaches can effectively capture heterogeneous patterns in complex biological datasets.^31^, ^32^ Of particular interest, Simple Logistic, despite its comparatively simple linear structure, performed at a level statistically comparable to that of the leading ensemble models. This finding suggests that the discriminatory information contained in the integrated morphological, color, and biochemical variables was sufficiently structured to be captured by different learning strategies rather than being dependent on a single model architecture.

Taken together, these results indicate that both ensemble methods and linear models can provide effective classification for multimodal agricultural datasets. However, classification success varied among genotypes. In particular, the recurrent misclassification between G2 and G3 suggests greater similarity between these genotypes within the evaluated feature space. Their overlap may reflect similarities in the integrated information available to the models, including individual‐seed morphological and color traits and experimental‐unit‐level biochemical variables. Similar challenges associated with phenotypic heterogeneity and inter‐class similarity have been reported in previous genotype characterization studies.^33^, ^34^ Specifically, while research on guava germplasm indicated that morpho‐biochemical variation complicates genotypic differentiation,^33^ studies on *Avena* species similarly reported that approaches relying solely on a single feature group remain insufficient.^34^

Furthermore, the interpretation of cross‐classifications in confusion matrix analyses has been emphasized as essential for understanding inter‐class overlap.^35^ The findings of the present study align closely with these reports. The mutual misclassifications observed between G2 and G3 are consistent with partial overlap in their measured phenotypic profiles, illustrating the difficulty of separating closely related classes within the evaluated feature space.

The present findings indicate that integrating morphological, color, and biochemical variables provides a multidimensional representation of chia seed phenotypes for genotype classification. The contribution of variables from different feature groups further suggests that complementary phenotypic information was utilized by the evaluated models.

Biochemical parameters emerged as informative variables for genotype differentiation. Feature importance and permutation importance analyses showed that protein, fiber, ash, and fat received relatively high importance scores in several of the evaluated models. Specifically, the distinct biochemical profile observed for G1 may have contributed to the comparatively high classification performance achieved for this genotype. Previous studies have likewise reported genotype‐dependent variation in protein composition, fatty acid profiles, and nutritional quality traits in chia.^1^, ^36^, ^37^ Malik and Riar^36^ reported variation in protein and fat profiles among chia genotypes, while Vera‐Cespedes^37^ showed that physicochemical and nutritional properties may also differ among genotypes. Furthermore, computational approaches have been used to identify biological variation associated with seed quality traits.^19^ The fact that several ML models assigned relatively high importance to similar biochemical parameters suggests that these compositional traits contributed useful information to genotype discrimination in the present dataset. Because these biochemical variables were measured at the experimental‐unit rather than individual‐seed level, their importance should be interpreted as evidence of compositional differences among the corresponding samples and not as direct biochemical discrimination among individual seeds. Consequently, beyond their established relevance to nutritional quality, these biochemical components may also represent informative compositional traits for chia genotype characterization.

Alongside these compositional differences, image‐derived morphological and color traits also contributed to genotype discrimination. In particular, hue, saturation, roundness, geometric mean diameter, and shape index received relatively high importance scores across multiple models, suggesting that seed morphology and color characteristics provided complementary information for distinguishing the evaluated genotypes. Morphometric descriptors have been shown to be useful for quantifying biological variation in plant phenotyping studies.^38^ Research on soybean and other seed species has similarly demonstrated the value of image‐derived color and shape characteristics for describing genotype‐associated variation.^39^, ^40^

Specifically, while Kim *et al*.^40^ demonstrated that image‐analysis‐based morphological characteristics possess strong discriminative potential for differentiating soybean genotypes, Baek *et al*.^39^ emphasized that such image‐based approaches offer substantial operational advantages in modern phenotyping platforms. In addition, ML‐based image segmentation has been shown to enhance image quality and analytical accuracy in automated phenotyping systems.^41^ Color space‐based computational approaches serve as robust analytical tools for phenotypic differentiation of biological specimens.^42^

In the present study, differences in hue among genotypes reflected variation in seed surface coloration, while morphological descriptors such as geometric mean diameter and roundness captured differences in seed form. The appearance of these variables among the higher‐ranked features indicates that image‐derived color and morphological information contributed to genotype discrimination. Image‐based morphometric analyses have similarly been recognized as rapid, objective, and scalable tools in modern seed phenotyping platforms.^43^, ^44^ These findings indicate the potential utility of digital phenotyping as a rapid and scalable approach for seed characterization in plant breeding.

Explainable artificial intelligence (XAI) provided an additional layer of interpretation by identifying the variables most strongly associated with model predictions. In the present study, feature importance and permutation importance analyses showed that several biochemical, morphological, and color‐related variables were repeatedly ranked among the more influential features across different algorithms. This consistency suggests that genotype discrimination was associated with multiple complementary phenotypic dimensions. However, these importance measures reflect associations with model predictions and should not be interpreted as evidence of causal biological relationships.

The value of XAI approaches for improving the transparency and interpretability of ML models in plant phenotyping has also been emphasized in recent studies.^45^, ^46^, ^47^ More broadly, explainable ML is receiving increasing attention in sustainable agriculture and data‐driven phenotyping, particularly where understanding the variables underlying model predictions is important.^48^, ^49^ In the present study, the recurrence of similar variables among the higher‐ranked features across different models provided a more transparent view of the classification process and helped identify phenotypic characteristics associated with chia genotype discrimination.

The findings of the present study indicate that classification models can serve not only as predictive tools but also as interpretable analytical approaches for examining phenotypic variables associated with genotype discrimination. The methodological novelty of this study lies in integrating image‐based morphological features, color parameters, and biochemical data using explainable ML methods. Such multimodal chia phenotyping studies remain limited in the literature. The use of repeated cross‐validation and multiple performance metrics provided a comprehensive assessment of model performance. However, the fact that the models were trained under controlled conditions and with a limited number of genotypes constrains their generalizability across diverse environmental contexts. Consequently, future studies should validate model performance utilizing datasets that encompass broader genetic populations and varied environmental conditions. In future studies, integrating hyperspectral imaging with deep learning‐based multimodal approaches may enable more detailed modeling of morphological and biochemical variation.^50^, ^51^ Similarly, UAV‐based multi‐sensor data fusion and deep learning approaches provide substantial operational advantages in automated phenotyping systems.^52^ Modern phenotyping platforms supported by computational models have begun to play a critical role in next‐generation plant breeding approaches.^53^ Overall, the present study shows that integrated image‐derived and biochemical information can provide an effective and interpretable framework for chia genotype classification, while broader genetic and environmental validation will be necessary before extending the approach to more diverse breeding or seed‐quality applications.^54^

## CONCLUSIONS

This study showed that morphological, color‐based, and biochemical information can be integrated effectively for the classification of chia genotypes. XGBoost, Simple Logistic, and RF achieved the strongest overall performance, with no statistically significant differences among these three models. The comparable performance of Simple Logistic further indicated that the discriminatory information contained in the integrated feature set could be captured by different learning strategies, including a relatively simple linear model. At the genotype level, G1 was consistently distinguished with high classification performance, whereas G2 and G3 showed greater overlap and were more frequently misclassified. Feature importance and permutation importance analyses highlighted several biochemical variables, particularly protein, fiber, ash, and fat, together with image‐derived color and morphological features such as hue, saturation, and shape index. Because the biochemical variables were measured at the experimental‐unit level, their contribution should be interpreted as compositional information associated with the corresponding seed samples rather than as biochemical measurements of individual seeds. Overall, the findings indicate that integrating image‐derived and biochemical information with interpretable ML provides an effective framework for chia genotype classification. Further validation across broader genetic materials and environmental conditions will be necessary to assess its potential for high‐throughput seed phenotyping and breeding applications.

## AUTHOR CONTRIBUTIONS

**Sevim Akcura:** Conceptualization, Data curation, Formal analysis, Methodology, Software, Visualization, Writing – original draft preparation. **Irem Poyraz:** Methodology, Formal analysis, Software, Visualization, Writing – original draft preparation. **Beyza Ciftçi:** Methodology, Formal analysis, Software, Visualization, Writing – original draft preparation. **Mahmut Kaplan:** Conceptualization, Data curation, Formal analysis, Methodology, Software, Visualization, Supervision, Writing – original draft preparation.

## CONFLICT OF INTEREST

The authors confirm that there are no competing interests, financial or otherwise, that could be perceived as having influenced the outcomes or interpretation of this study.

## Supporting information


**Table S1.** Overall performance metrics of machine learning algorithms for chia seed genotype classification.
**Table S2.** Class‐level performance metrics of machine learning algorithms.

## Data Availability

The data supporting the findings of this study are available from the corresponding author upon reasonable request.
