# Supplementary material for: Image‐based and biochemical multimodal phenotyping for explainable classification of chia (Salvia hispanica L.) genotypes
Source: J Sci Food Agric. 2026 Aug 31;107(1):980–95. doi: 10.1002/jsfa.71038 (PMC13756570; doi:10.1002/jsfa.71038)
Supplement: Supplementary file 1 [file JSFA-107-980-s001.docx]

**Table S1.** Overall performance metrics of machine learning algorithms for chia seed genotype classification

| **Parameters** | **Algorithm** | | | | | | | | | |
| --- | --- | --- | --- | --- | --- | --- | --- | --- | --- | --- |
|  | **RF** | **SL** | **XGBoost** | **SMO-SVM** | **GB** | **ET** | **MLP** | **kNN** | **DT** | **AdaBoost** |
| Accuracy | 0.868 | 0.867 | 0.865 | 0.854 | 0.852 | 0.850 | 0.844 | 0.815 | 0.722 | 0.710 |
| Kappa statistic | 0.823 | 0.822 | 0.820 | 0.806 | 0.802 | 0.800 | 0.792 | 0.753 | 0.629 | 0.613 |
| Mean absolute error | 0.173 | 0.094 | 0.091 | 0.112 | 0.102 | 0.190 | 0.137 | 0.144 | 0.139 | 0.367 |
| Root mean squared error | 0.254 | 0.222 | 0.220 | 0.232 | 0.227 | 0.266 | 0.242 | 0.266 | 0.373 | 0.424 |
| Relative absolute error (%) | 46.22 | 25.17 | 24.20 | 29.83 | 27.14 | 50.77 | 36.50 | 38.34 | 37.11 | 97.76 |
| Root relative squared error (%) | 58.76 | 51.19 | 50.79 | 53.65 | 52.35 | 61.43 | 55.90 | 61.34 | 86.15 | 97.81 |
| TP | 1041 | 1040 | 1038 | 1025 | 1022 | 1020 | 1013 | 978 | 866 | 852 |
| TN | 3441 | 3440 | 3438 | 3425 | 3422 | 3420 | 3413 | 3378 | 3266 | 3252 |
| FP | 159 | 160 | 162 | 175 | 178 | 180 | 187 | 222 | 334 | 348 |
| FN | 159 | 160 | 162 | 175 | 178 | 180 | 187 | 222 | 334 | 348 |
| Precision | 0.867 | 0.867 | 0.864 | 0.854 | 0.853 | 0.849 | 0.843 | 0.822 | 0.721 | 0.729 |
| Recall | 0.868 | 0.867 | 0.865 | 0.854 | 0.852 | 0.850 | 0.844 | 0.815 | 0.722 | 0.710 |
| F-Measure | 0.867 | 0.867 | 0.865 | 0.854 | 0.852 | 0.849 | 0.843 | 0.813 | 0.721 | 0.714 |
| MCC | 0.823 | 0.822 | 0.820 | 0.806 | 0.802 | 0.801 | 0.793 | 0.757 | 0.629 | 0.616 |
| ROC Area | 0.971 | 0.974 | 0.975 | 0.969 | 0.972 | 0.971 | 0.969 | 0.946 | 0.814 | 0.865 |
| PRC Area | 0.924 | 0.933 | 0.933 | 0.922 | 0.927 | 0.924 | 0.918 | 0.876 | 0.598 | 0.670 |

*Overall classification performance metrics of ten machine learning algorithms, including accuracy, kappa statistic, precision, recall, F-measure, MCC, ROC area, and PRC area, obtained from repeated 10-fold cross-validation experiments. TP, true positive; TN, true negative; FP, false positive; FN, false negative. RF, Random Forest; SL, Simple Logistic; XGBoost, eXtreme Gradient Boosting; SMO-SVM, Support Vector Machine with sequential minimal optimization; GB, Gradient Boosting; ET, Extra Trees; MLP, Multilayer Perceptron; kNN, k-Nearest Neighbors; DT, Decision Tree.*

**Table S2.** Class-level performance metrics of machine learning algorithms

| **Algorithm** | **Class** | **TP** | **TN** | **FP** | **FN** | **Precision** | **Recall** | **F-Measure** | **MCC** | **ROC Area** | **PRC Area** |
| --- | --- | --- | --- | --- | --- | --- | --- | --- | --- | --- | --- |
| Random Forest | G1 | 2876 | 8827 | 173 | 124 | 0.943 | 0.959 | 0.951 | 0.934 | 0.997 | 0.991 |
| Random Forest | G2 | 2458 | 8568 | 432 | 542 | 0.851 | 0.819 | 0.835 | 0.781 | 0.966 | 0.919 |
| Random Forest | G3 | 2407 | 8436 | 564 | 593 | 0.810 | 0.802 | 0.806 | 0.742 | 0.953 | 0.867 |
| Random Forest | G4 | 2573 | 8483 | 517 | 427 | 0.833 | 0.858 | 0.845 | 0.792 | 0.970 | 0.920 |
| Simple Logistic | G1 | 2864 | 8867 | 133 | 136 | 0.956 | 0.955 | 0.955 | 0.940 | 0.996 | 0.988 |
| Simple Logistic | G2 | 2444 | 8561 | 439 | 556 | 0.848 | 0.815 | 0.831 | 0.776 | 0.967 | 0.919 |
| Simple Logistic | G3 | 2505 | 8514 | 486 | 495 | 0.838 | 0.835 | 0.836 | 0.782 | 0.961 | 0.910 |
| Simple Logistic | G4 | 2609 | 8480 | 520 | 391 | 0.834 | 0.870 | 0.851 | 0.801 | 0.973 | 0.920 |
| XGBoost | G1 | 2873 | 8852 | 148 | 127 | 0.951 | 0.958 | 0.954 | 0.939 | 0.997 | 0.991 |
| XGBoost | G2 | 2506 | 8601 | 399 | 494 | 0.863 | 0.835 | 0.849 | 0.800 | 0.970 | 0.928 |
| XGBoost | G3 | 2452 | 8473 | 527 | 548 | 0.823 | 0.817 | 0.820 | 0.761 | 0.960 | 0.890 |
| XGBoost | G4 | 2608 | 8513 | 487 | 392 | 0.843 | 0.869 | 0.856 | 0.807 | 0.975 | 0.928 |
| SMO-SVM | G1 | 2829 | 8797 | 203 | 171 | 0.933 | 0.943 | 0.938 | 0.917 | 0.994 | 0.981 |
| SMO-SVM | G2 | 2470 | 8550 | 450 | 530 | 0.846 | 0.823 | 0.834 | 0.780 | 0.966 | 0.912 |
| SMO-SVM | G3 | 2393 | 8431 | 569 | 607 | 0.808 | 0.798 | 0.803 | 0.738 | 0.946 | 0.874 |
| SMO-SVM | G4 | 2564 | 8478 | 522 | 436 | 0.831 | 0.855 | 0.843 | 0.789 | 0.973 | 0.928 |
| Gradient Boosting | G1 | 2811 | 8840 | 160 | 189 | 0.946 | 0.937 | 0.942 | 0.922 | 0.996 | 0.988 |
| Gradient Boosting | G2 | 2437 | 8603 | 397 | 563 | 0.860 | 0.812 | 0.835 | 0.783 | 0.968 | 0.923 |
| Gradient Boosting | G3 | 2445 | 8362 | 638 | 555 | 0.793 | 0.815 | 0.804 | 0.737 | 0.956 | 0.881 |
| Gradient Boosting | G4 | 2600 | 8488 | 512 | 400 | 0.835 | 0.867 | 0.851 | 0.800 | 0.973 | 0.926 |
| Extra Trees | G1 | 2919 | 8740 | 260 | 81 | 0.918 | 0.973 | 0.945 | 0.926 | 0.997 | 0.991 |
| Extra Trees | G2 | 2434 | 8606 | 394 | 566 | 0.861 | 0.811 | 0.835 | 0.783 | 0.964 | 0.919 |
| Extra Trees | G3 | 2316 | 8521 | 479 | 684 | 0.829 | 0.772 | 0.799 | 0.736 | 0.950 | 0.864 |
| Extra Trees | G4 | 2610 | 8412 | 588 | 390 | 0.816 | 0.870 | 0.842 | 0.788 | 0.972 | 0.923 |
| MLP | G1 | 2882 | 8722 | 278 | 118 | 0.912 | 0.961 | 0.936 | 0.914 | 0.995 | 0.985 |
| MLP | G2 | 2438 | 8539 | 461 | 562 | 0.841 | 0.813 | 0.827 | 0.770 | 0.966 | 0.922 |
| MLP | G3 | 2324 | 8521 | 479 | 676 | 0.829 | 0.775 | 0.801 | 0.738 | 0.953 | 0.871 |
| MLP | G4 | 2614 | 8476 | 524 | 386 | 0.833 | 0.871 | 0.852 | 0.801 | 0.973 | 0.925 |
| kNN | G1 | 2871 | 8645 | 355 | 129 | 0.890 | 0.957 | 0.922 | 0.896 | 0.993 | 0.974 |
| kNN | G2 | 2083 | 8757 | 243 | 917 | 0.896 | 0.694 | 0.782 | 0.731 | 0.927 | 0.868 |
| kNN | G3 | 2097 | 8355 | 645 | 903 | 0.765 | 0.699 | 0.730 | 0.647 | 0.912 | 0.797 |
| kNN | G4 | 2708 | 8002 | 998 | 292 | 0.731 | 0.903 | 0.808 | 0.742 | 0.957 | 0.868 |
| Decision Tree | G1 | 2630 | 8622 | 378 | 370 | 0.874 | 0.877 | 0.875 | 0.834 | 0.917 | 0.797 |
| Decision Tree | G2 | 2033 | 8058 | 942 | 967 | 0.683 | 0.678 | 0.681 | 0.575 | 0.787 | 0.544 |
| Decision Tree | G3 | 1908 | 7875 | 1125 | 1092 | 0.629 | 0.636 | 0.633 | 0.509 | 0.756 | 0.491 |
| Decision Tree | G4 | 2105 | 8121 | 879 | 895 | 0.705 | 0.702 | 0.704 | 0.605 | 0.802 | 0.570 |
| AdaBoost | G1 | 1998 | 8912 | 88 | 1002 | 0.958 | 0.666 | 0.786 | 0.750 | 0.990 | 0.969 |
| AdaBoost | G2 | 1968 | 7904 | 1096 | 1032 | 0.642 | 0.656 | 0.649 | 0.531 | 0.873 | 0.662 |
| AdaBoost | G3 | 2076 | 7723 | 1277 | 924 | 0.619 | 0.692 | 0.654 | 0.531 | 0.704 | 0.376 |
| AdaBoost | G4 | 2293 | 7796 | 1204 | 707 | 0.656 | 0.764 | 0.706 | 0.601 | 0.893 | 0.663 |

*Class-level (genotype-specific) classification performance of ten machine learning algorithms across the four chia seed genotype classes (G1–G4), obtained from repeated 10-fold cross-validation. Metrics include precision, recall, F-measure, Matthews correlation coefficient (MCC), ROC area, and PRC area. TP, true positive; TN, true negative; FP, false positive; FN, false negative. Algorithms are ordered by overall performance (highest to lowest).*
